# Characterization of a Novel Hot-Spring Cyanobacterium *Leptodesmis sichuanensis* sp. Nov. and Genomic Insights of Molecular Adaptations Into Its Habitat

**DOI:** 10.3389/fmicb.2021.739625

**Published:** 2022-01-28

**Authors:** Jie Tang, Lian-Ming Du, Meijin Li, Dan Yao, Ying Jiang, Malgorzata Waleron, Krzysztof Waleron, Maurycy Daroch

**Affiliations:** ^1^Antibiotics Research and Re-Evaluation Key Laboratory of Sichuan Province, Sichuan Industrial Institute of Antibiotics, Chengdu University, Chengdu, China; ^2^School of Environment and Energy, Peking University Shenzhen Graduate School, Shenzhen, China; ^3^Laboratory of Plant Protection and Biotechnology, Intercollegiate Faculty of Biotechnology, University of Gdańsk and Medical University of Gdańsk, University of Gdańsk, Gdańsk, Poland; ^4^Department of Pharmaceutical Microbiology, Faculty of Pharmacy Medical University of Gdańsk, Gdańsk, Poland

**Keywords:** *Leptodesmis*, 16S rRNA, genome, comparative genomics, hot spring, thermophilic cyanobacterium, adaptation, Leptolyngbyaceae

## Abstract

The newly described genus *Leptodesmis* comprises several strains of filamentous cyanobacteria from diverse, primarily cold, habitats. Here, we sequenced the complete genome of a novel hot-spring strain, *Leptodesmis* sp. PKUAC-SCTA121 (hereafter A121), isolated from Erdaoqiao hot springs (pH 6.32, 40.8°C), China. The analyses of 16S rRNA/16S-23S ITS phylogenies, secondary structures, and morphology strongly support strain A121 as a new species within *Leptodesmis, Leptodesmis sichuanensis* sp. nov. Notably, strain A121 is the first thermophilic representative of genus *Leptodesmis* and more broadly the first *Leptodesmis* sp. to have its genome sequenced. In addition, results of genome-scale phylogenetic analysis and average nucleotide/amino acid identity as well as *in silico* DNA-DNA hybridization and patristic analysis verify the establishment of genus *Leptodesmis* previously cryptic to *Phormidesmis*. Comparative genomic analyses reveal that the *Leptodesmis* A121 and *Thermoleptolyngbya sichuanensis* A183 from the same hot-spring biome exhibit different genome structures but similar functional classifications of protein-coding genes. Although the core molecular components of photosynthesis, metabolism, and signal transduction were shared by the two strains, distinct genes associated with photosynthesis and signal transduction were identified, indicating that different strategies might be used by these strains to adapt to that specific niche. Furthermore, the complete genome of strain A121 provides the first insight into the genomic features of genus *Leptodesmis* and lays the foundation for future global ecogenomic and geogenomic studies.

## Introduction

Thermophilic cyanobacteria are unique photoautotrophic microorganisms that can survive and thrive in adverse, thermal environments (Elleuche et al., 2015) with optimum growth and metabolic activities at high temperatures with the support of highly stable enzymes (Patel et al., 2019). Thus, they exhibit considerable biotechnological potential, e.g., CO_2_ sequestration and production of biofuels, bioactive compounds, and pigments (Liang et al., 2019; Patel et al., 2019).

Despite the abundance of thermal ecosystems around the world, the diversity of thermophilic cyanobacteria is still highly underestimated. Next-generation sequencing (NGS) is extensively employed to investigate the cyanobacterial diversity in thermal environments in recent years (Tang et al., 2018b; Alcorta et al., 2020; Chen et al., 2021). However, NGS results often provide ample abstract data, e.g., operational taxonomic units (OTUs), higher taxonomic groups. Reports on novel isolates of thermophilic cyanobacteria are scarce. Isolating these microrganisms from different ecosystems is crucial for multidisciplinary studies and provides potential strains and genetic sequence data for biotechnology and industrial applications. Further, isolated strains provide opportunity for in-depth study studies to understand the various characteristics, such as morphology, genomics, and adaptation (Cordeiro et al., 2020; Tang et al., 2021).

*Leptodesmis* is a newly described genus based on environmental, morphological, and molecular data (Raabova et al., 2019). Two species in this genus have been studied, *L. alaskaensis* (Strunecky et al., 2020) and *L. paradoxa* (Raabova et al., 2019). In our previous study, we isolated a *Leptodesmis* strain, A121, from an Erdaoqiao hot spring, Sichuan, China (Tang et al., 2018a). Strain A121 can grow at 50°C and/or at the concentration of 0.1 M NaHCO_3_ (Tang et al., 2018a).

To date, there has been no genome sequence available for any of the *Leptodesmis* strains. To fill this gap and reveal thermal adaptations, we sequenced the entire genome of A121 in this study. This strain was also characterized using molecular and morphological analysis, and further comparative genomics was conducted to reveal the genome structures and adaptation mechanisms of the *Leptodesmis* sp. A121 and *Thermoleptolyngbya sichuanensis* A183 from the same hot-spring biome. This study provides a foundation for future ecogenomic and geogenomic studies on global thermophilic cyanobacteria.

## Materials and Methods

### Strain Information and Genome Sequencing

The strain A121 was originally isolated by Dr. Md. Mahfuzur R. Shah from Erdaoqiao hot springs (pH 6.32, 40.8°C) in Ganzi Prefecture of Sichuan Province, China, as previously described (Tang et al., 2018b). Unicyanobacterial culture of A121 was used to establish experimental cultures essentially as described previously (Tang et al., 2018a). The strain was initially denoted and deposited in the Peking University Algae Collection as PKUAC-SCTA121 and has also been deposited in the Freshwater Algae Culture Collection at the Institute of Hydrobiology (FACHB-collection) with accession number FACHB-3319. Cultivation of the strain and basic physiological studies closely follow previously published material (Tang et al., 2021). In short, the strain was grown in BG11 shake-flasks in at 45°C, 100 rpm, photoperiod 16 L: 8D (45 μmol m^–2^ s^–1^).

The short reads of A121 were sequenced using Illumina NovaSeq PE150 at the Beijing Novogene Bioinformatics Technology Co., Ltd. Illumina PCR adapter reads and low-quality reads from the paired ends were filtered by the step of quality control using the company’s own compiling pipeline. In total, 495,688,800 bp data were generated. For Oxford Nanopore Technologies (ONT) sequencing, sequencing libraries were generated using SQK-LSK109 Kit following the manufacturer’s recommendations and protocol. The long reads were sequenced using a PromethION sequencer (Oxford Nanopore Technologies, Oxford, United Kingdom), and as a result, a total of 1,792,271,158 bp for A121 was generated. The raw data from Nanopore sequencing was converted from fast5 to fastq using Albacore software in the MinKNOW package. The generated circular contig was error corrected with a Geneious Prime (2020.2.2) mapper^®^ to generate the final genome draft.

Gene prediction and annotation were performed using the NCBI prokaryotic genome annotation pipeline and further using the RAST annotation system to minimize poor calls. The circular plot of the A121 genome was generated in Circos v0.68 (Krzywinski et al., 2009). Default settings were used for all bioinformatics tools unless stated otherwise.

### Phylogeny

Sequences of the 16S rRNA gene were collected for A121 by extraction from the genome sequence and for cyanobacterial references through BLAST search. Muscle implemented in Mega7 (Kumar et al., 2016) was employed to generate multiple sequence alignments, and manual editing was carried out when necessary. Maximum-likelihood (ML) inference of 16S rRNA sequences was reconstructed using PhyML v3.3 (Guindon et al., 2010). Parameter settings in PhyML were followed as described (Tang et al., 2019).

To infer the phylogenomic relationship, genomes of A121 and 13 focus taxa (nine representatives from family Leptolyngbyaceae and three from family Oculatellaceae as references and one from family Synechococcaceae as the outgroup) were included for analysis. The quality of the genomes was evaluated using CheckM (Parks et al., 2015) to ensure a high-quality data set with high completeness (≥ 95%) and low contamination (< 5%). Single-copy genes shared by all genomes were extracted from the homologous gene clusters identified by OrthoMCL (Li et al., 2003) and concatenated employing custom Perl script. Multisequence alignment was performed using MAFFT v7.453 (Standley, 2013). The supergene alignment was subjected to phylogenomic inference using IQ-TREE v2.1.3 (Minh et al., 2020). The optimal substitution model for phylogenomic analysis was selected from 546 protein models by ModelFinder implemented in IQ-TREE. Bootstrap tests (1,000 replicates) were conducted for evaluation of tree topologies using UltraFast Bootstrap (Hoang et al., 2018).

Multi Locus Sequence Analysis encompassing 14 loci (11,869 bp): *cpc*A (465 bp), *cpc*B (486 bp), *cpc*H (204 bp), *gyr*A (597 bp), *kai*A (408 bp), *kai*B (303 bp), *kai*C (1,475 bp), *nir* (1,457 bp), *rbc*C (318 bp), *rbc*L (1,241 bp), *rec*A (672 bp), *rec*N (1,513 bp), *rpo*C (689 bp), *sec*A (2,039 bp) was performed using MEGA X software^1^ using the ML method and the GTR + G + I with Gamma distribution as the best nucleotide substitution model. The bootstrap consensus was inferred from 1,000 replicates. Phylogenetic analysis was carried out using 34 strains on the basis of sequences retrieved from genomes of *Leptodesmis* and closely related Leptolyngbyaceae strains that genomic or good quality metagenomic data were available at GenBank (in June 2021).

### Analysis of 16S-23S ITS

The 16S-23S ITS region was extracted from the A121 genome, and cyanobacterial sequences were retrieved from GenBank through BLAST search as references. Phylogenetic analysis of 16S-23S ITS was performed using the aforementioned pipeline of 16S rRNA analysis. For the strains ascribable to *Leptodesmis*, the conserved domains of the 16S-23S ITS region: D1-D1′, D2, D3, boxA, and D4; and its variable regions (V2, boxB, and V3) were identified as previously described (Iteman et al., 2000). The tRNAs presented in the spacer were identified by tRNAscan-SE v1.3.1 (Lowe and Eddy, 1997). The secondary structures of the identified fragments were individually determined by Mfold web server (Zuker, 2003). Except for the use of the structure draw mode untangle with loop fix, default conditions in Mfold were used in all cases.

### Morphology Investigation

Strain A121 was inspected at 400 × magnification using light microscopy (LM, DP72, OLYMPUS, Japan) equipped with an image acquisition system (U-TV0.63XC, OLYMPUS, Japan). Microscopic investigations were also conducted using scanning electron microscopy (SEM) (SU8100, HITACHI, Japan) and transmission electron microscopy (TEM) (HT7800, HITACHI, Japan). All microscopic operation was performed following the procedures as described by Tang et al. (2021).

### Genome Analysis

Pairwise genome comparisons were performed to calculate average nucleotide identity (ANI) and average amino acid identity (AAI) using the ANI/AAI calculator (Rodriguez-R and Konstantinidis, 2016). Further, clustering analysis was conducted for genomes based on AAI distances using the unweighted pair group method with algorithmic mean (UPGMA).

*In silico* DDH comparison was performed employing the genome-to-genome distance calculator (GGDC 2.0)^2^ (Tang et al., 2021) using the recommended BLAST + alignment and formula 2 (identities/HSP length) (Johansen et al., 2011). Patristic distances between and within the *Leptodesmis* sp. A121 and other Leptolyngbyaceae strains were calculated with PATRISTICv1.0 software (Zhang et al., 2019) using concatenated sequences of 14 loci: *cpc*B, *cpc*A, *cpc*H, *gyr*A, *kai*A, *kai*B, *kai*C, *nir*, *rbc*C, *rbc*L, *rec*A, *rec*N, *rpo*C, *sec*A.

Comparative genomics was performed between *Leptodesmis* sp. A121 (CP075171) and *T. sichuanensis* A183 (CP053661). The two strains originated from the same sampling site (Tang et al., 2018a). We speculated that similar molecular adaptations of the two strains were shaped or evolutionarily driven by the thermal ecosystem. The homologous genes between genomes were identified by OrthoMCL (Li et al., 2003). The customized Venn diagram was drawn using an online tool to exhibit the orthologous and unique genes between the strains. The EggNOG database v4 (Jaime et al., 2016) was used for functional classification of protein sequences using the following thresholds: *E*-value cutoff of 1E-6, ≥ 30% identity and 70% coverage. Pairwise genome alignments were performed using MUMmer v3.2.3 (Kurtz et al., 2004).

## Results and Discussion

### Complete Genome Sequence of *Leptodesmis* sp. A121

**FIGURE 1:**
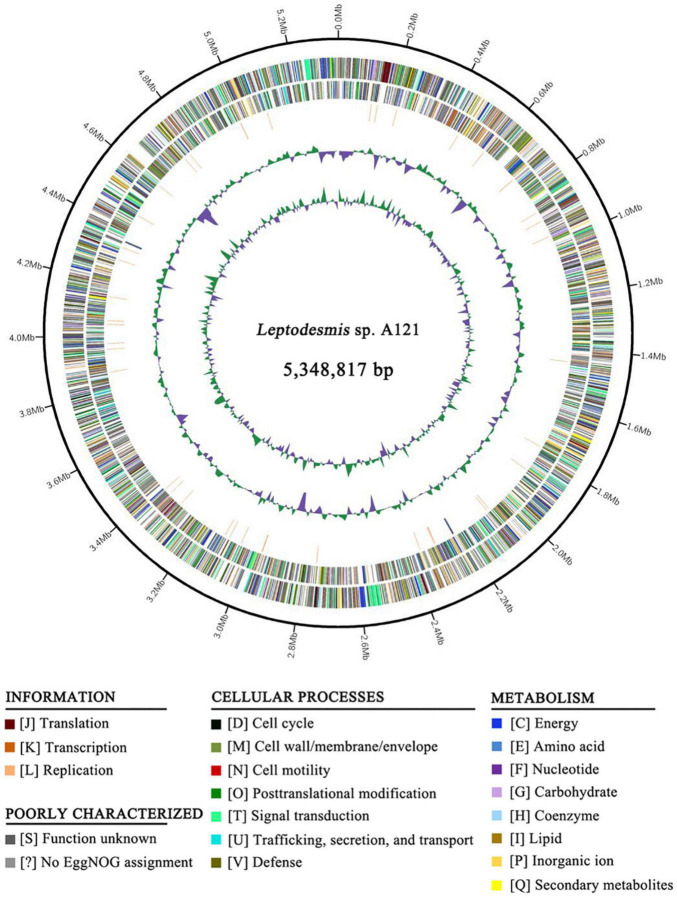
Genome illustration of *Leptodesmis* sp. A121. Rings are as follows (outer–inner): CDS on plus strand; CDS on minus strand; rRNA (orange) and tRNA (blue); the last two circles represent GC content and GC skew both calculated for a 10 kb window with 1 kb stepping. The CDS are color-coded by functional categories of EggNOG database.

Whole-genome sequencing of *Leptodesmis* sp. A121 generated a total of 3,304,592 filtered paired-end reads (clean data), providing approximately 93-fold coverage of the genome. Bioinformatic assembly obtained a single circular chromosome with a size of 5,348,817 bp (GC content, 50.32%) and no plasmid. The annotation of the A121 genome indicated two ribosomal RNA operons, 45 tRNA genes, and 5345 protein-coding genes (Figure 1 and Supplementary Table 1). Approximately 52.6% of protein-coding genes were identified as hypothetical proteins (Supplementary Table 2). No strong patterns of GC-skew were observed in the chromosome organization of A121 (Figure 1). This result was similar to other hot-spring cyanobacteria, e.g., *Thermosynechococcus elongatus* BP-1 (Yasukazu et al., 2002) or *T. sichuanensis* A183 (Tang et al., 2021).

### Phylogenetic Reconstruction of 16S rRNA Gene

**FIGURE 2:**
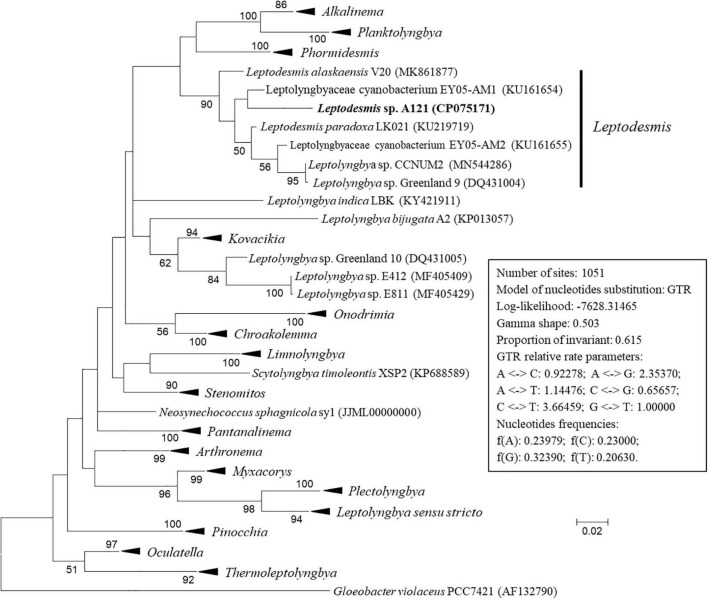
Maximum-likelihood phylogenetic tree of 16S rRNA gene sequences. Branches of strains affiliated to the same genus were collapsed and referred by black triangle, followed by the genus name. The complete tree is shown in Supplementary Figure 1. Only bootstrap values > 50% are indicated at nodes. Scale bar = 2% substitutions per site.

The ML phylogenetic tree (Figure 2 and Supplementary Figure 1) inferred by 16S rRNA gene sequences categorized the cyanobacterial strains into 17 well-supported genera within the family Leptolyngbyaceae and two genera within family Oculatellaceae and *Gloeobacter* as an outgroup. In addition, the tree indicates that five *Leptolyngbya* strains, strain Greenland 10, E412, E811, A2 and LBK, were quite divergent from the genus *Leptolyngbya sensu stricto* and could be classified into three new genera. The actual taxonomic delineation of these *Leptolyngbya* strains must be carefully investigated using polyphasic approaches (e.g., more molecular markers, morphology, etc.) in a separate study because genus *Leptolyngbya* has been recognized as polyphyletic (Johansen et al., 2011).

Strain A121 clustered with six strains, forming a well-defined clade of genus *Leptodesmis*. Of the six strains, *L. alaskaensis* V20 (Strunecky et al., 2020) and *L. paradoxa* LK021 (Raabova et al., 2019) are two recently proposed species; *Leptolyngbya* sp. Greenland 9 (Roeselers et al., 2007) and CCNUM2 (Zhang et al., 2019) were probably misclassified as indicated by the clear conflict between phylogeny and taxonomy; strain EY05-AM1 and EY05-AM2 were taxonomically unassigned and just labeled as Leptolyngbyaceae cyanobacterium. The 16S rRNA sequence identities between A121 and the six strains ranged from 94.9 to 96.9% (Table 1), suggesting A121 as a novel species within *Leptodesmis* based on the recommended threshold of 16S rRNA gene identity for bacterial species (98–99%) or genera (94.5–95%) demarcation (Rodriguez-R et al., 2018). Strain Greenland 9 and CCNUM2 can be proposed to be another new species within *Leptodesmis* based on the 16S rRNA phylogeny and identity (Figure 2 and Table 1), but the affiliation of strain EY05-AM1 and EY05-AM2 to the same species as Greenland 9 and CCNUM2 was not strongly supported in light of the 16S rRNA identity values (97.6–98.3%). Besides this, the shorter length of 16S rRNA sequence of strain EY05-AM1 and EY05-AM2 (Table 1) might affect the identity value and phylogenetic positions on the tree of the current study. Therefore, the actual taxonomy of strain EY05-AM1 and EY05-AM2 should be thoroughly studied in the future.

**TABLE 1:** Sequence identities (%) of 16S rRNA gene of *Leptodesmis* strains studied.

Strain	A121	V20	LK021	CCNUM2	Greenland 9	EY05-AM1	EY05-AM2
*Leptodesmis* sp. A121	100 (1,453)						
*Leptodesmis alaskaensis* V20	96.1 (1,147)	100 (1,145)					
*Leptodesmis paradoxa* LK021	94.9 (1,148)	95.6 (1,149)	100 (1,140)				
*Leptolyngbya* sp. CCNUM2	96.5 (1,420)	96.0 (1,115)	96.5 (1,116)	100 (1,419)			
*Leptolyngbya* sp. Greenland 9	96.6 (1,425)	96.1 (1,120)	96.6 (1,101)	99.9 (1,419)	100 (1,425)		
Leptolyngbyaceae cyanobacterium EY05-AM1	96.9 (1,147)	96.8 (1,149)	96.4 (1,149)	97.6 (1,114)	97.7 (1,120)	100 (1,147)	
Leptolyngbyaceae cyanobacterium EY05-AM2	95.8 (1,110)	96.7 (1,110)	96.4 (1,110)	98.3 (1,110)	98.2 (1,110)	97.4 (1,110)	100 (1,110)

*The alignment length (bp) is indicated inside brackets.*

Intriguingly, strain A121 (Tang et al., 2018a) and Greenland 9 (Roeselers et al., 2007) were originally isolated from hot springs in sharp contrast to habitat niches of strain V20 (Strunecky et al., 2020) and LK021 (Raabova et al., 2019) recovered from polar regions. The distinct habitat niches suggest that these strains might be different ecotypes. Strain CCNUM2 appeared to be an intermediate ecotype between thermophiles and psychrophiles because it was isolated from humid moss on forest limestone where temperature ranges from 29 to 39°C in the summer to 1–8°C in the winter (Zhang et al., 2019). Strain EY05-AM1 and EY05-AM2 originated from a wet waterfall wall in El Yunque national forest, Puerto Rico. The acclimation of *Leptodesmis* strains to diverse habitats strongly implies the underlying genetic diversity within this genus. Verifying this speculation will be an interesting topic using phylogenomic and ecogenomic approaches with the help of more genome sequences of this genus. Unfortunately, to date there is only one genome sequence available for genus *Leptodesmis*, that presented in this study, strain A121.

### Phylogeny and Secondary Structures of 16S-23S ITS

**FIGURE 3:**
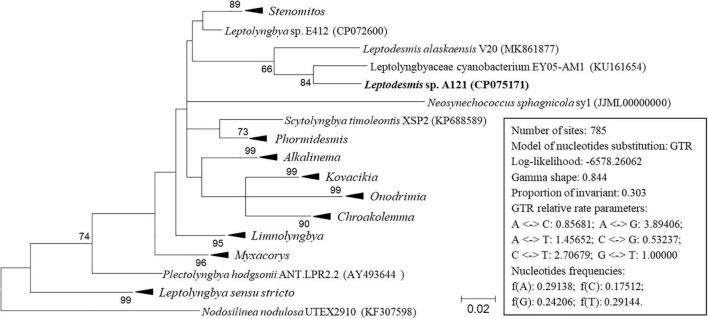
ML phylogenetic tree of 16S-23S ITS sequences. Branches of strains affiliated to the same genus are collapsed and referred by black triangle, followed by the genus name. The complete tree is shown in Supplementary Figure 2. Only bootstrap values > 50% are indicated at nodes. Scale bar = 2% substitutions per site.

In addition to the 16S rRNA gene, the 16S-23S ITS region is another commonly used molecular marker for delineating cyanobacterial ecotypes or species (Brito et al., 2017). In light of sequence unavailability, the sequence data set of 16S-23S ITS is smaller than that of 16S rRNA. Although the phylogenetic inference of 16S-23S ITS (Figure 3 and Supplementary Figure 2) show a distinct topology from that of the 16S rRNA gene, the classification of species was consistent at the genus level. The three strains ascribable to *Leptodesmis* were placed into a well-separated clade but showed evident genetic divergence as indicated by branch length (Figure 3), suggesting that the three strains are different species within *Leptodesmis*. However, 16S-23S ITS sequences are extraordinarily divergent and probably bring about misleading taxonomic assignment without secondary structure domain analysis (Johansen et al., 2011). Therefore, secondary structure analyses of 16S-23S ITS were also carried out as an essential complement to further resolve the taxonomy within *Leptodesmis*.

Unfortunately, only strain A121 and EY05-AM1 had available full-length ITS sequences, whereas strain V20 had partial sequence (Table 2). Excluding two highly conserved tRNAs from ITS sequences, the length of the remaining ITS sequences were 325 and 236 bp for strain A121 and EY05-AM1, respectively (Table 2). Among the three *Leptodesmis* strains, identical sequences were observed in conserved domains D3 (GGTTT), whereas conserved domain D2 exhibited three sequence types with one-nucleotide difference, CTTTCAAACTA in A121, CTTTCAAACTT in V20 and CTTTCAAGCTA in EY05-AM1. Strain A121 and EY05-AM1 both showed identical sequences of conserved domain boxA (GGACCTTGAAAA) and D4 (CTGCATA).

**TABLE 2:** The length (bp) summary of regions within16S-23S ITS of *Leptodesmis* strains studied.

Strain	ITS length (tRNA removed)	D1-D1′ helix	D2	D3	V2 helix	Boxb helix	Boxa	D4	V3 helix
*Leptodesmis* sp. A121	325	63	12	5	81	33	12	7	98
Leptolyngbyaceae cyanobacterium EY05-AM1	236	62	12	5	4	33	12	7	44
*Leptodesmis alaskaensis* V20	NA	77	12	5	4	NA	NA	NA	NA

*NA, not available.*

**FIGURE 4:**
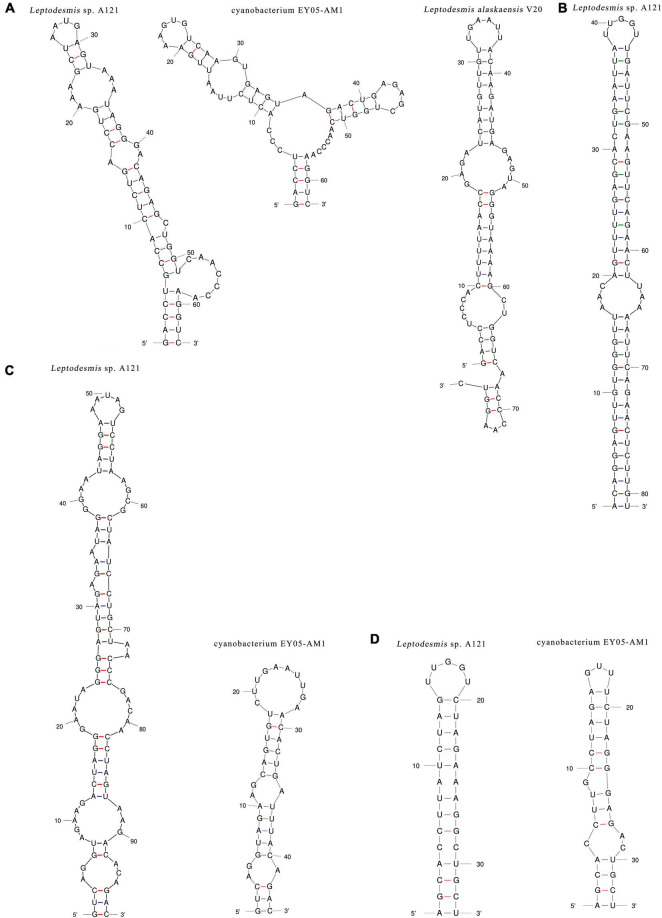
Hypothetical secondary structures of D1-D1′ helix **(A)**, V2 helix **(B)**, V3 helix **(C),** and boxB **(D)** of 16S-23S ITS of *Leptodesmis* strains.

The inferred D1-D1′ helices (Figure 4A) of the three *Leptodesmis* strains showed different lengths (62–77 bp) and distinct structures to each other. The V2 helix of strain A121 was 81 residues long (Figure 4B), whereas that of strain EY05-AM1 and V20 only contained four residues, resulting in no secondary structure predicted. Strain A121 and EY05-AM1 shared a basal stem structure (GUC-GAC) of V3 helices, but the considerable difference in length of V3 helices generated distinct structures (Figure 4C). Although strain A121 showed the same residue length (33 bp) of boxB helix to EY05-AM1, the structures of boxB helix (Figure 4D) were clearly incompatible with each other.

In summary, the phylogeny and secondary structures undoubtedly differentiate A121 from the other *Leptodesmis* strains, confirming the delineation of the A121 strain as a new species of *Leptodesmis*. The secondary structure analysis of the variable regions (D1-D1′, V2, boxB, and V3 helices) proved to be an effective approach for species-level identification within *Leptodesmis*. Although the V2 helix was the most inconsistent (Table 2), it was the least taxonomic informative in light of its extreme variability and absence in some cyanobacterial strains (Sciuto and Moro, 2016). In addition, more sequences are essential for accurately resolving the other *Leptodesmis* strains included in the 16S rRNA tree.

### Morphological Features of *Leptodesmis* sp. A121

**FIGURE 5:**
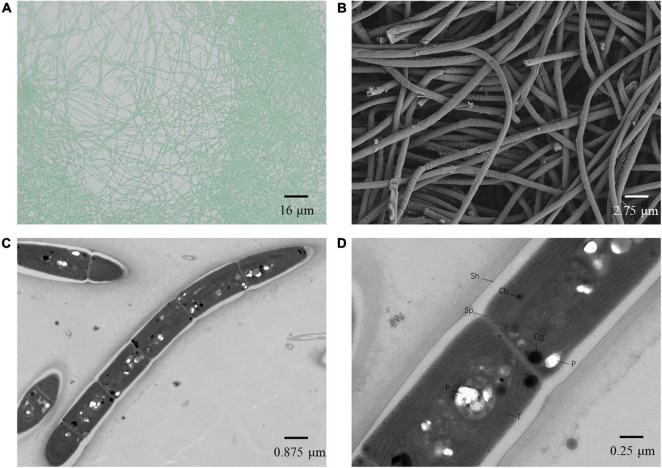
Microscopic images of *Leptodesmis* sp. A121. **(A)** light microscopy image. **(B)** SEM image. **(C,D)** TEM images. Cb, carboxysome; Cg, cyanophycin granule; P, polyphosphate body; Sh, sheath; Sp, septum; T, thylakoid membrane. Magnifications were 400× **(A)**, 5,000× **(B)**, 8,000× **(C),** and 12,000× **(D)**.

Light microscopy (Figure 5A) indicated straight, wavy, curved, and occasionally bent trichomes of strain A121. The SEM indicated long, thin, unbranched filaments, whereas the TEM (Figures 5B,C) showed that some shorter trichomes of A121 are comprised of 10–25 individual barrel-shaped cells. The trichomes observed in the longitudinal direction were solitary filaments composed of long cylindrical cells with contraction in the transverse wall (Figure 5B). In addition, TEM revealed the ultrastructure of A121, a single cell with a length of 2.0–3.0 μm and a width of 0.9–1.4 μm (Figures 5C,D). The long cylindrical cells were separated by the centripetal invagination of the cell wall, and no intracellular connection between the vegetative cells was observed (Figure 5D). The TEM analysis also showed that the three to five thylakoid layers were located in parallel at the inner periphery of the cells (Figures 5C,D). Sheaths, septum, carboxysomes, phycocyanin granules, and polyphosphate bodies were present in the cytoplasm (Figures 5C,D). The trichomes were surrounded by a clearly visible layer of extracellular polymeric substances (EPS).

Comparisons of morphological features of known *Leptodesmis* strains are summarized in Table 3. In general, the three *Leptodesmis* strains were blue-green and were straight, curved, flexuous, or wavy, solitary filaments with one sheath of one cell. Dissimilarities, however, were also observed. Strains A121 and *L. alaskaensis* were motile, whereas *L. paradoxa* was immotile. The cell width of *L. paradoxa* was considerably wider than that of A121 and *L. alaskaensis*. A broader range of cell lengths was noticed in *L. alaskaensis*, whereas A121 exhibited a medium cell length. Meanwhile, the CCNUM2 strain showed moderate cell widths and lengths compared with other representatives of *Leptodesmis*.

**TABLE 3:** Comparison of morphological features of *Leptodesmis* strains.

Strain	Isolation source	Filaments	Motility	Cell width (μm)	Cell length (μm)	Sheaths	Number of thylakoids	Color of trichomes	References
*Leptodesmis* sp. A121	Green mat, hot spring, Sichuan, China	Straight, curved, flexuous or wavy, solitary, one filament per sheath dark green	Motile	0.9–1.4	2.0–3.0	Thick, colorless, Multilayered	3–5	Blue-green	This study
*Leptodesmis alaskaensis* V20	Green mat in dry channels, Lake NE2 in Toolik lake area, Alaska, United States	Straight, curved, flexuous or wavy, solitary, one filament per sheath pale-blue green to dark green	Motile	1–1.5	1.4–4.3	Thick, colorless	NA	Pale blue-green to green	Strunecky et al., 2020
*Leptodesmis paradoxa* LK021	Mud in pool, Deception Island, Antarctica	Straight, curved, flexuous or wavy, solitary, one filament per sheath pale blue-green to dark green	None	2.5–3.5	1.0–1.5	Thick, colorless	NA	Pale blue green	Raabova et al., 2019
*Leptolyngbya* sp. CCNUM2	Humid moss on forest limestone, Wuhan, China	Non-branched	NA	1.57–1.71	1.38–1.96	NA	NA	NA	Zhang et al., 2019

*NA, not available.*

### Phylogenomic and Genomic Distance Analysis

**FIGURE 6:**
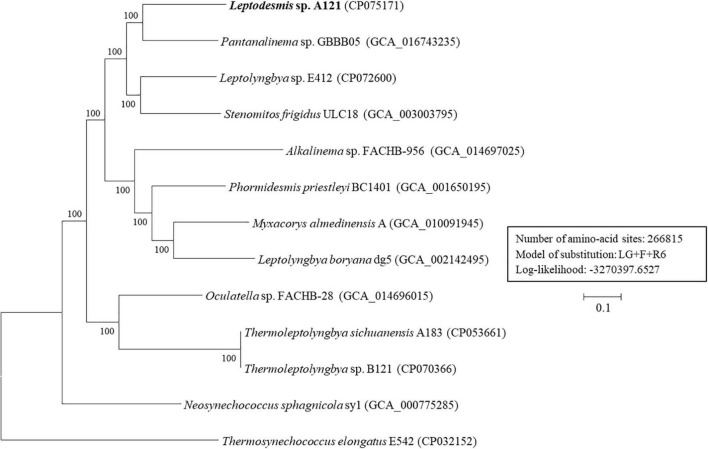
ML phylogenomic tree of concatenated protein alignment of 845 single-copy genes shared by all genomes. Bootstrap values are indicated at nodes. Scale bar = 10% substitutions per site.

To further resolve relationships between *Leptodesmis* strains and related taxa, phylogenomic and genome distances were compared. Analysis of homologous gene clusters indicated 845 single-copy genes present in the 13 strains studied. The concatenated alignments of these genes exhibited 266,815 aligned amino acid sites. ML inference of the supergene alignment generated a phylogeny with strong bootstrap support (100%) for all branches (Figure 6), clearly defining each genus. This phylogenomic topology was almost consistent with that of the 16S rRNA gene (Figure 2). There was only one exception. The phylogenetic analysis of the 16S rRNA gene clearly indicated the affiliation of *Neosynechococcus sphagnicola* sy1 to the family Leptolyngbyaceae, whereas sy1 was placed in an ambiguous position from the genome-scale phylogeny. Further studies are required to ascertain the actual taxonomy of this strain using, e.g., polyphasic approaches. Multilocus sequence analysis (MLSA) of 14 loci from 34 strains (Supplementary Figure 3) further support the findings of the previous phylogenomic and 16S rRNA gene analyses. To summarize, the phylogenomic trees supported the new delineation of *Leptodesmis*, which was a cryptic genus of *Phormidesmis* (Raabova et al., 2019).

Results of genome-wide ANI and AAI (Supplementary Figure 4) all conformed to the suggested values for species (ANI > 96%, AAI ≥ 95%) and genus (ANI < 83%, AAI ≤ 70%) delimitation (Walter et al., 2017; Jain and Rodriguez, 2018), respectively. The UPGMA clustering based on genome-scale AAI distances (Supplementary Figure 4) was similar to the molecular phylogenies. To shed more light on global sequence similarity between *Leptodesmis* sp. A121 and different Leptolyngbyaceae, combined *in silico* DNA-DNA hybridization (Supplementary Table 3) and patristic distance (Supplementary Table 4) analyses were performed. The estimated *is*DDH values dropped below 32% when the genome of *Leptodesmis* sp. A121 was compared with other Leptolyngbyaceae strains that were used as queries (Supplementary Table 3). When patristic distances were compared between *Leptodesmis* sp. A121 and other Leptolyngbyaceae strains, significantly higher values of 0.24–0.35 were noticed (Supplementary Table 4). Thus, the results indicate that there are no other representatives described until now, which might belong or be assigned to *Leptodesmis* based on genomic data deposited in the public databases. These findings combined further reinforce that *Leptodesmis* was correctly delineated as a separate genus from *Phormidesmis.* Meanwhile, within the *Leptodesmis* genus, the strain A121 could be delineated as a separate species based on currently available sequence information.

### Genome Comparison

To elucidate the genomic features of *Leptodesmis* sp. A121 and *T. sichuanensis* A183 isolated from the same Erdaoqiao hot spring, a comparative analysis was performed. Genome characteristics and related information for the two hot-spring strains were shown in Supplementary Table 1. Venn diagram (Supplementary Figure 5) indicated that 3056 genes were shared by the two genomes, and 2177 genes were present only in the *Leptodesmis* sp. A121 genome. Functional classification showed that the distribution patterns of EggNOG categories (Supplementary Figure 6) were similar between the two genomes. Pairwise genome alignments (Supplementary Figure 7) suggest considerably low conservation in chromosomal organization between the two genomes. These results indicate that the two strains from the same ecosystem showed different genome structures but similar functional classifications of protein-coding genes.

### Photosynthesis

The core sets of genes coding for both photosystem I and photosystem II were conserved in genomes of the two hot-spring strains. However, these genes also exhibit variations in distribution and copies. For example, two copies of cytochrome c-550 genes (*psbV1* and *psbV2*) were tandemly clustered in the *Leptodesmis* genome while separately arranged in the *Thermoleptolyngbya* genome. The two genomes contained *psbA* genes (*psbA1*, *psbA2*, and *psbA3*) encoding the reaction center D1 protein of photosystem II, but the copies of *psbA* genes were three for the *Thermoleptolyngbya* genome and four for the *Leptodesmis* genome. For the auxiliary components in the light energy transmission system of photosynthesis, homologous genes encoding allophycocyanin were present in the *Leptodesmis* genome, while there were allophycocyanin and phycocyanin in the *Thermoleptolyngbya* genome. The different types of phycobilisome proteins between the two hot-spring strains indicate that an alternative light-absorbing strategy was utilized for acclimation to the same environments, possibly enabling a degree of their stratification within the biofilm.

Essential genes required for the carbon-dioxide concentrating mechanism (CCM) were also conserved between the two hot-spring strains. However, some variations were found in the components of CCM-related genes. The arrangements of *ndhA*-*M* genes encoding the NADPH dehydrogenase (NDH-1) complexes responsible for uptake of gaseous CO_2_ systems (Ogawa and Mi, 2007) were completely distinct between genomes. Eleven copies of *Hat/HatR* gene encoding high-affinity carbon uptake protein were present in the *Leptodesmis* genome and 13 in the *Thermoleptolyngbya* genome. For the bicarbonate transport system, *bicA1*, a low-affinity Na^+^-dependent bicarbonate transporter gene was present in the *Leptodesmis* genome, whereas both *bicA1* and *bicA2* were present in the *Thermoleptolyngbya* genome. The *cmpABCD* operon encoding high-affinity ABC-type bicarbonate transport system was present in both genomes. In contrast, high-affinity Na^+^-dependent bicarbonate transport family permease genes, *sbtA* and *sbtB*, were present only in the *Thermoleptolyngbya* genome. The different systems of carbon assimilation in the two strains might be flexibly activated to cater to the varying demands of carbon uptake in light of an economy of regulation from a survival aspect. Therefore, the increased genetic repertoire of bicarbonate transporters can indicate A183 strain’s preference for this source of inorganic carbon vs. A183 (Tang et al., 2018a).

Interestingly, the flavodoxin gene (*fldA*) was not found in the *Leptodesmis* genome but present the *Thermoleptolyngbya* genome. Under iron-deficient conditions, flavodoxins can substitute ferredoxin to function as an electron transfer, and the iron stress-inducible proteins (*isiA*) increase the light-absorbing efficiency of PSI in the form of PSI-*isiA*-flavodoxin supercomplex (Cao et al., 2020). Thus, this kind of energy and electron transfer is not the prevailing acclimation and regulation mechanism for the *Leptodesmis* strain to overcome usually iron-deficient natural environments. In addition, the *isiA* gene in the *Leptodesmis* genome showed a significant degree of similarity to that of mesophilic *Synechocystis* sp. PCC6803 (37.3% amino acid sequence identity). Further study is required to elucidate the physiological significance of structural differences.

### Metabolism

The ability to utilize various organic and inorganic sources of substances is vital for hot-spring strains to survive in oligotrophic aquatic environments (Esteves-Ferreira et al., 2018). The core metabolism genes were shared by genomes of the two hot-spring strains. The strains are likely able to import and utilize nitrate and urea as nitrogen sources, in light of hosting complete gene sets of *nrtABCD* (nitrate assimilation), *urtABCDE* (ABC-type urea transport system) and *ureA*, -*BC*, *-EF*, -*G* (urease genes). The *Leptodesmis* genome contained genes encoding for nitrogenases (*nif*) similar to those in the *Thermoleptolyngbya* genome, suggesting that the *Leptodesmis* strain is a nitrogen-fixing cyanobacteria. The ability of nitrogen fixation was experimentally confirmed by nitrogenase activity (Supplementary Figure 8). Moreover, the two genomes possessed homologs associated with ammonium transporter, glutamine synthetase and glutamine amidotransferase, and nitrogen assimilation transcriptional activator, which played roles in ammonium ion assimilation (Bolay et al., 2018) and nitrogen control for efficient utilization of intracellular resources responding to ambient low nitrogen (Vega-Palas et al., 2010), respectively.

Hot-spring strains are usually capable of sulfur assimilation as many geothermal systems possess abundant sulfur. The ABC-type sulfate transport system (the sulfate-thiosulfate permease, *sulT*) was present in the two genomes. However, the distribution of these coding genes varied significantly. In the *Leptodesmis* genome, two of the four genes encoding the subunits of *sulT* formed a *cysTW* operon, and the other two genes (*cysA*, *cysP*) were individually arranged away from the operon. By contrast, the *Thermoleptolyngbya* genome comprised a *cysPTW* operon and *cysA* located far away from the operon. In addition, the *Leptodesmis* genome harbored three distinct copies of sulfate permease encoded by *sulP*, whereas the *Thermoleptolyngbya* genome contained one. The high-affinity ModABC molybdate transport system encoded by the *modABC* operon was present in the two genomes and can be also used for sulfur transport.

Two distinct sets of gene clusters for the phosphate-specific transport system (encoded by *pstSCAB* operon) were detected in the *Leptodesmis*, whereas the *Thermoleptolyngbya* genome hosted only one operon. One *pstSCAB* operon of the *Leptodesmis* genome shared high amino acid sequence similarity with that of the *Thermoleptolyngbya* genome. The other one was quite distinct, indicating that this operon might be obtained from different origins through horizontal gene transfer. An arsenical resistance operon (glyceraldehyde-3-phosphate dehydrogenase, MFS-type efflux pump *arsJ*, arsenical-resistance protein ACR3) with an upstream repressor was present in the two genomes. This result indicated that the gene operon may confer regulable arsenate resistance to the two strains (Chen et al., 2016). Overall, the *Leptodesmis* and *Thermoleptolyngbya* strains appeared to have similar metabolism systems to acclimate to the shifting conditions in hot-spring environments.

### Signal Transduction

The perception of environmental stress and the subsequent transduction of stress signals are conducted through the two-component regulatory systems in cyanobacteria (Wuichet and Zhulin, 2010). In the *Leptodesmis* genome, 32 and 42 genes were identified as potential genes encoding histidine kinases and response regulators, respectively. However, the association between histidine kinases and response regulators cannot directly be elucidated due to the scattered distribution of these genes in the genome. This is in sharp contrast to several cases in prokaryotes with which a single two-component system was tandemly arranged into operons or closely clustered (Wuichet et al., 2010; Aguilar et al., 2014). Most of the histidine kinases and response regulators were conserved between the two genomes, but variations also existed. For example, the *Leptodesmis* genome only had a complete set of motility-related two-component signaling systems, including *cheA*, methyl-accepting chemotaxis protein, *cheW* and *cheY*, whereas the *Thermoleptolyngbya* genome had two sets. In addition, the GGDEF/EAL domain proteins in the genomes varied in gene numbers from 7 to 17, suggesting the complexity of cyclic-di-GMP signaling pathways might be different between the two strains.

### Other Features in the *Leptodesmis* Genome

The Ni-Fe bidirectional hydrogenase enzyme (encoded by *hoxEFUYH*) was present in the *Leptodesmis* genome but absent in the *Thermoleptolyngbya* genome. The *hoxW* gene, involved in subunit assembly of *hoxH*, was located downstream the *hoxEFUYH* around 5.5 kb in the *Leptodesmis* genome. In addition, the two genomes hosted polyketide synthase (PKS) module-related proteins, a family of multidomain enzymes that produce polyketides (a large class of secondary metabolites) (Maurya et al., 2019). The genes encoding acyl-[acyl-carrier-protein] (ACP) reductase and aldehyde decarbonylase were tandemly arranged in the *Leptodesmis* genome but scattered in the *Thermoleptolyngbya* genome. These genes are involved in the biosynthesis of pentadecane and heptadecane alkanes that are the major constituents of various fuels, such as diesel, jet fuel, and gasoline (Peralta-Yahya et al., 2012). The *Leptodesmis* genome comprised genes encoding Rpn family recombination-promoting nuclease/putative transposase that was absent in the *Thermoleptolyngbya* genomes. The Rpn family can promote the acquisition process of obtaining new genes from other bacteria to adapt to ecological niches and survive under stressful conditions (Kingston and Ponkratz, 2017). Therefore, this result indicated that hot-spring strains may conduct gene regulation to accelerate the process of horizontal gene transfer. Furthermore, only the *Leptodesmis* genome contained VapC family toxin that belongs to type II toxin-antitoxin system and induces RNA cleavage (McKenzie et al., 2012). Further study may be required to elucidate the significance of these distinct genes.

## Conclusion

In this manuscript, the polyphasic approach allowed us to propose a new species, *Leptodesmis sichuanensis*, and the delineation of strain A121 as representative of this new taxon. This proposal was strongly supported by 16S rRNA/16S-23S ITS phylogenies, secondary structures, and morphology. Further, strain A121 is the first thermophilic representative of genus *Leptodesmis*. From the perspective of genomics, the results of phylogenomics, MLSA, ANI/AAI, *in silico* DNA-DNA hybridization and patristic distance assays reinforced the recent delineation of genus *Leptodesmis* from genus *Phormidesmis*. Comparative genomic analyses revealed that the *Leptodesmis* and *Thermoleptolyngbya* strains from the same hot-spring biome exhibited different genome structures but similar functional classifications of protein-coding genes. Although the core molecular components of photosynthesis, metabolism, and signal transduction were shared by the two strains, distinct features of genes were also noticed in terms of photosynthesis, and signal transduction, etc., indicating that different strategies might be used by these strains to adapt to the specific niche. In addition, the complete genome of strain A121 provides first insight into the genomic feature of genus *Leptodesmis* and lays foundation for future globally ecogenomic and geogenomic studies.

### Taxonomic Treatment and Description of *Leptodesmis sichuanensis* Daroch, Tang, and Shah et al. sp. nov.

The classification system that was applied was based on Komárek et al. (2014).

Taxon description in accordance with the prescriptions of the International Code of Nomenclature for Algae, Fungi and Plants (Shenzhen code) (Turland et al., 2018).

Phylum: Cyanobacteria

Order: Synechococcales

Family: Leptolyngbyaceae

*Description*: Observed under the light microscopy the filaments of A121 strain were blue-green, thin, straight, bent, wavy, and occasionally curved (Figure 5). Trichomes observed with TEM show barrel shaped cells forming a single filament without any branching; meanwhile, SEM and light microscopy photos indicate much longer filaments. Trichomes observed in longitudinal direction were characterized by long cylindrical cells with contraction in the transverse wall. Typical length of each cell ranged from 2.0 to 3.0 μm and had a width of 0.9–1.4 μm (Figure 5). Each of the cylindrical cells was separated from a neighboring cell with centripetal invagination of the cell wall, and no connections between the cells were observed. Each of the cells had between three and five thylakoid layers located in parallel on the inner periphery of the cells. Cells were observed to contain carboxysomes, cyanophycin granules, and polyphosphate bodies. The strain was motile. The strain was capable of growing in standard BG11 medium at a maximal temperature of 50°C and in the presence of 0.1 M NaHCO_3_. The strain can be successfully cryopreserved in 10% DMSO for a period exceeding 24 months.

Type strain: is A121 (= FACHB-3319).

*Type species*: *Leptodesmis sichuanensis Daroch, Tang, and Shah et al*. sp. nov. (see below).

*Etymology*: Species epithet derives from the name of the collection site.

*Type locality*: Ecology of type locality: the sample occurred as macroscopic dark green mat attached to the sinter around the pond with a small amount of mucilage around the entire mat. Sample collection was done in 12.05.2016 with the humidity being close to 71%. Ambient temperature at the time of collection was 15°C and the light intensity was around 1000 lux. The temperature of the hot spring, its pH, and concentration of total dissolved solids were, 40.8°C, 6.32, and 447 mmol L^–1^, respectively.

*Habitat*: thermal springs in Ganzi Prefecture of the spring was 6.32 and of Sichuan Province, China (30°05′14″N, 101°56′55″ E) *Leptodesmis* species. The strain is diazotrophic and exhibits experimentally verified nitrogenase activity with acetylene reduction assay, and the corresponding gene cluster has been identified in strains genome. Development of heterocysts was not observed.

*Holotype here designated*: the culture of *Leptodesmis sichuanensis Daroch, Tang et Shah* sp. *nov*. was initially denoted and deposited in Peking University Algae Collection as PKUAC-SCTA121 has also been deposited in the Freshwater Algae Culture Collection at the Institute of Hydrobiology (FACHB-collection) with accession number FACHB-3319 as *Leptodesmis* species after identification and authentication on the basis of the full-length sequencing of the 16S rRNA gene along with folding of the secondary structures of the 16S–23S ITS region. After proper identification and authentication, the culture is being maintained in the FACHB under the accession number FACHB-3391.

## Data Availability Statement

The names of the repository/repositories and accession number(s) can be found below: https://www.ncbi.nlm.nih.gov/genbank/, CP075171.

## Author Contributions

JT: conceptualization, methodology, validation, formal analysis, investigation, data curation, writing – original draft, writing – review, and editing, visualization, supervision, project administration, funding acquisition. L-MD: methodology, software, data curation. ML: formal analysis, investigation, data curation, writing – original draft. DY: formal analysis, investigation, data curation. YJ: data curation, writing – review and editing. MW and KW: methodology, data curation, formal analysis, visualization, writing – review, and editing. MD: conceptualization, methodology, resources, data curation, writing – original draft, writing – review and editing, supervision, project administration, funding acquisition. All authors contributed to the article and approved the submitted version.

## Conflict of Interest

The authors declare that the research was conducted in the absence of any commercial or financial relationships that could be construed as a potential conflict of interest.

## Publisher’s Note

All claims expressed in this article are solely those of the authors and do not necessarily represent those of their affiliated organizations, or those of the publisher, the editors and the reviewers. Any product that may be evaluated in this article, or claim that may be made by its manufacturer, is not guaranteed or endorsed by the publisher.

## Footnotes

1
www.megasoftware.net

2
http://ggdc.dsmz.de/distcalc2.php
