# Supplementary material for: Characterization of a Novel Hot-Spring Cyanobacterium Leptodesmis sichuanensis sp. Nov. and Genomic Insights of Molecular Adaptations Into Its Habitat
Source: Front Microbiol. 2022 Jan 28;12:739625. doi: 10.3389/fmicb.2021.739625 (PMC8832068; doi:10.3389/fmicb.2021.739625)
Supplement: Supplementary file 1 [file Data_Sheet_1.docx]

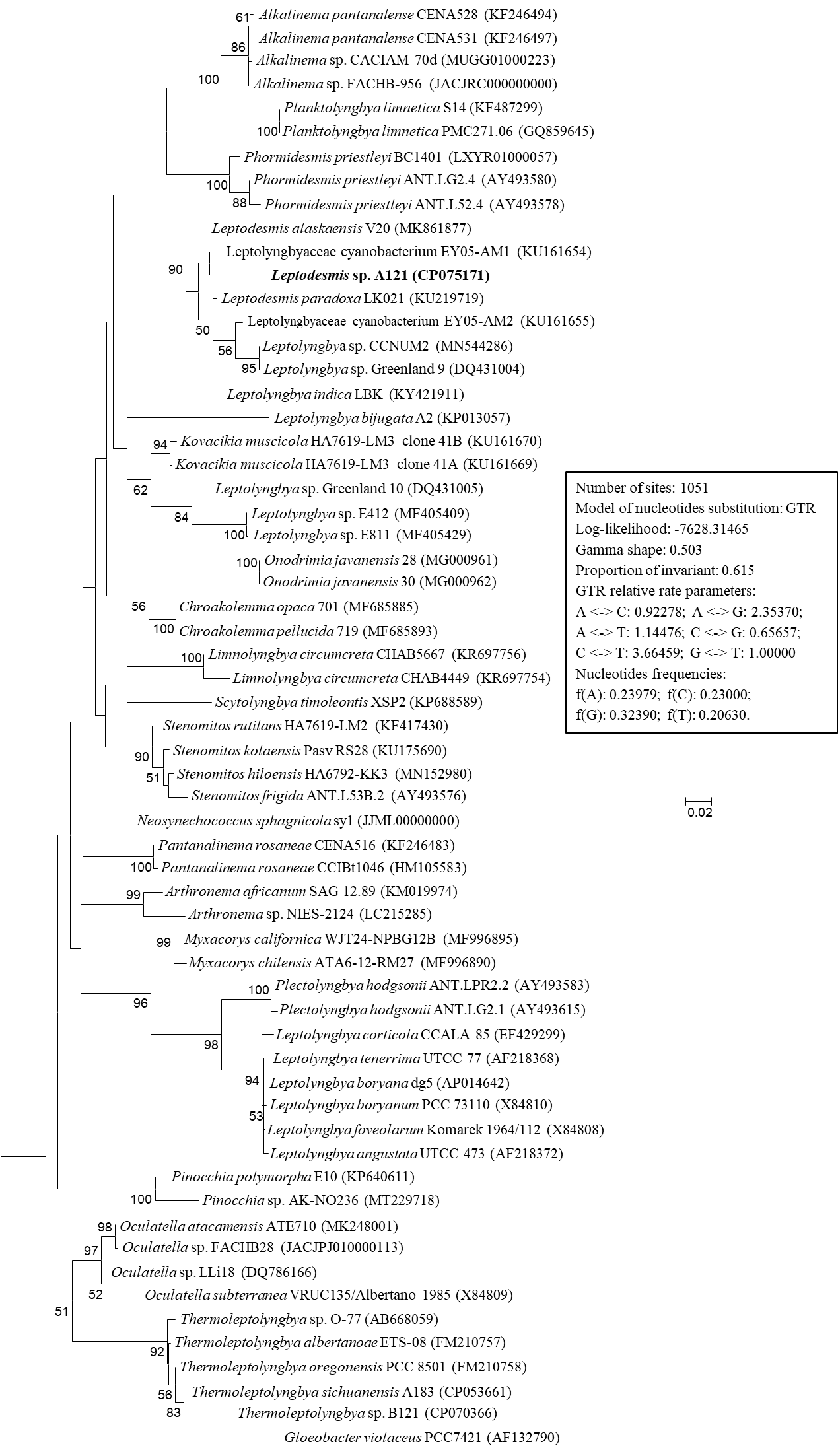


**Supplementary Figure 1** ML phylogenetic tree of 16S rRNA gene sequences. Strain no. in bold represent the strains identified in this study. Only bootstrap values>50% (1000 nonparametric replications) are indicated at nodes. Scale bar = 2% substitutions per site.

**
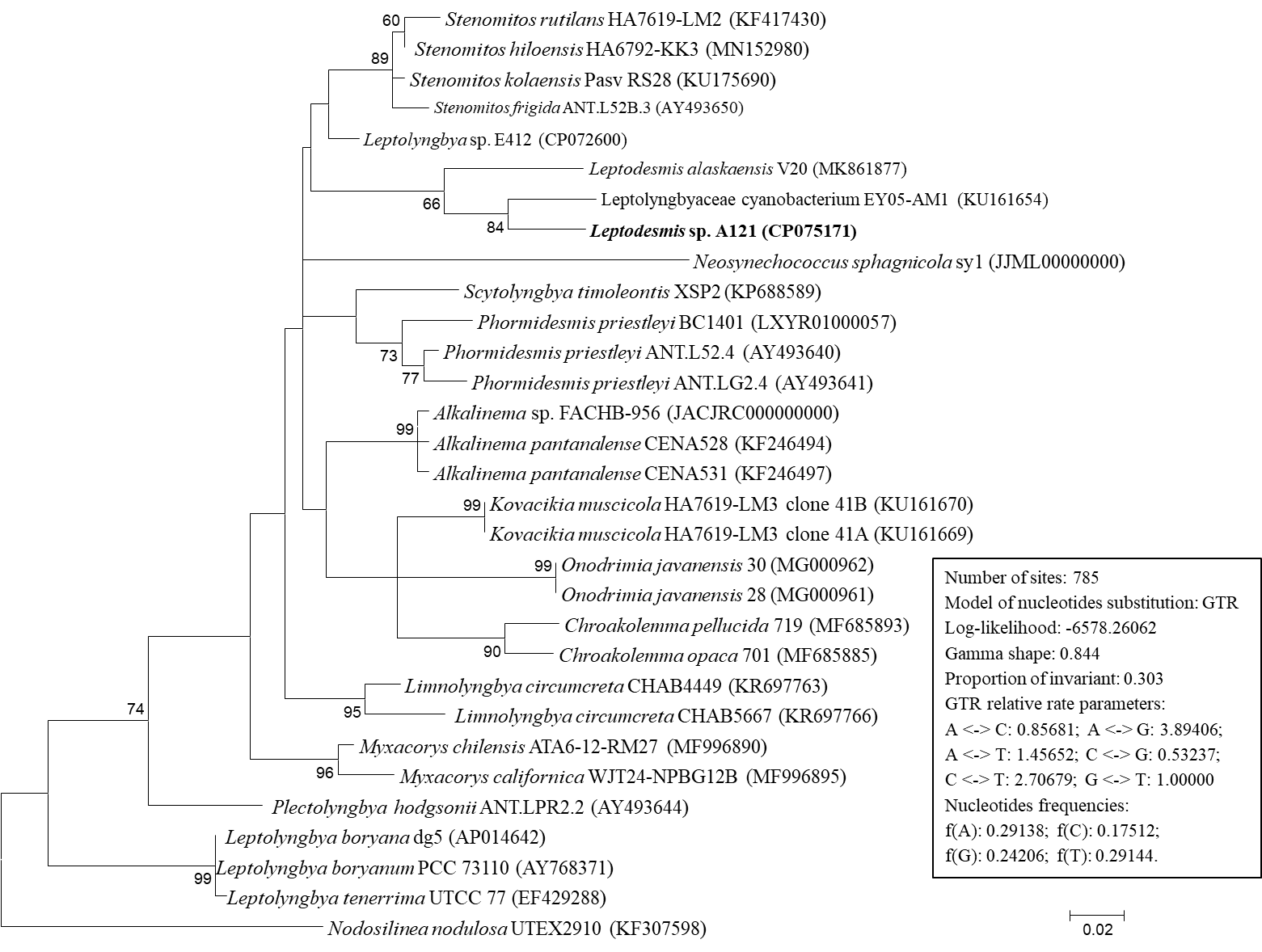
**

**Supplementary Figure 2** ML phylogenetic tree of 16S-23S ITS sequences. Strain no. in bold represent the strains identified in this study. Only bootstrap values>50% (1000 nonparametric replications) are indicated at nodes. Scale bar = 2% substitutions per site.


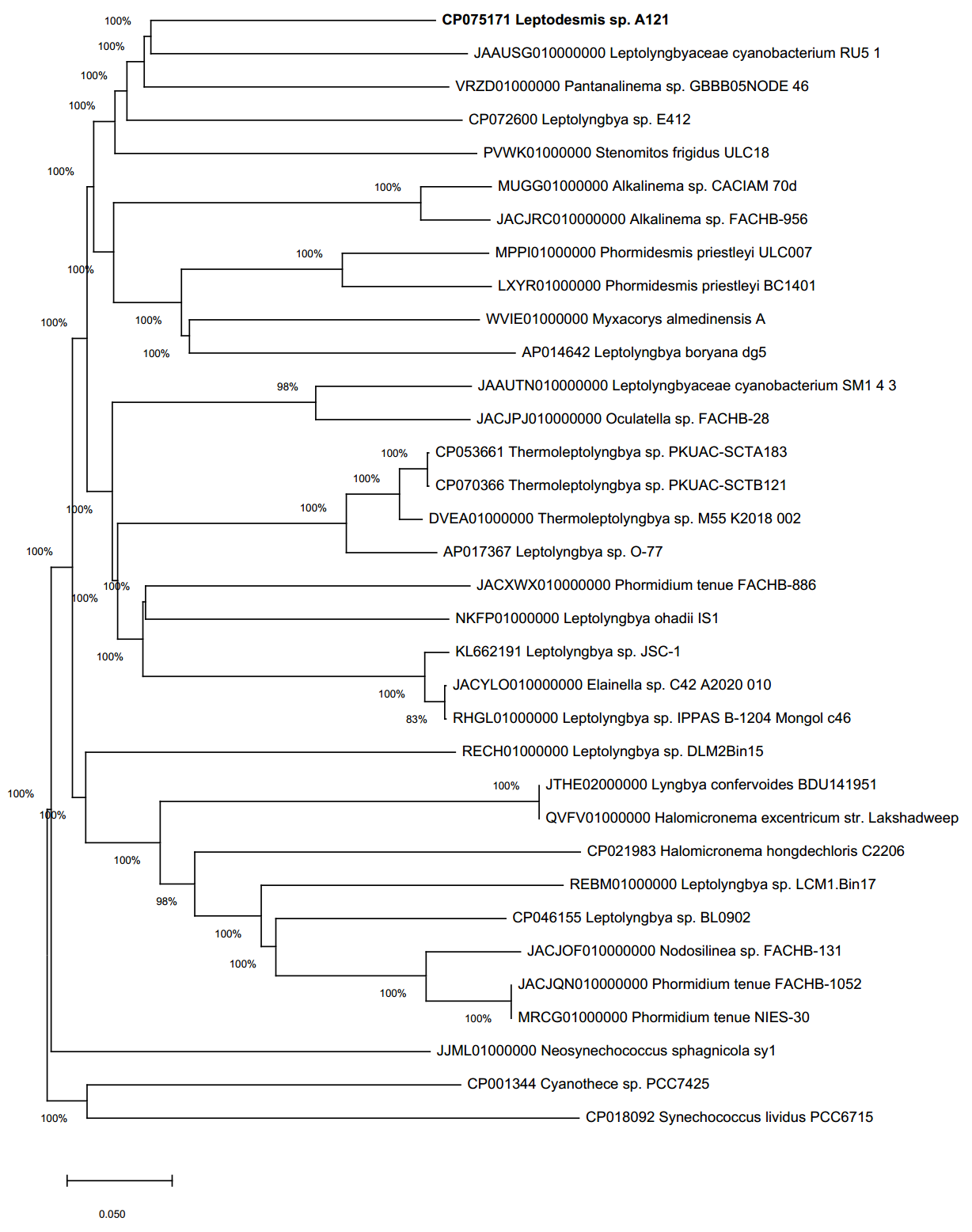


**Supplementary Figure 3** Maximum-Likelihood tree based on concatenated sequences of fourteen genes: *cpcA*, *cpcB*, *cpcH*, *GyrA*, *kaiA*, *kaiB*, *kaiC*, *Nir*, *rbcC*, *rbcL*, *recA*, *recN*, *rpoC*, *secA*


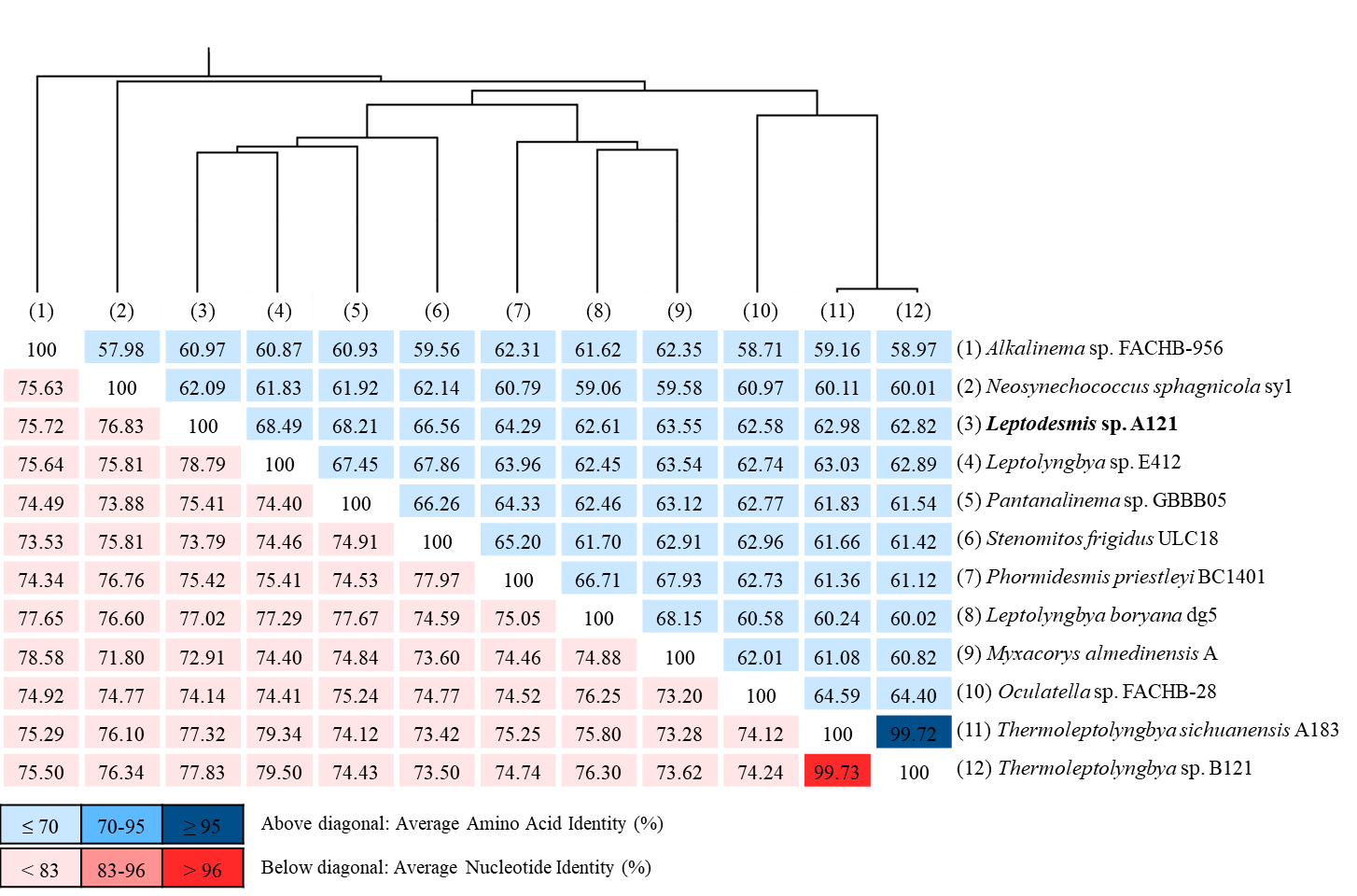


**Supplementary Figure 4** Values of ANI and AAI between cyanobacterial genomes. Similarity clustering of genomes was done by AAI distances using UPGMA method. The numbers above and below the diagonal indicate the AAI and ANI values (%), respectively.


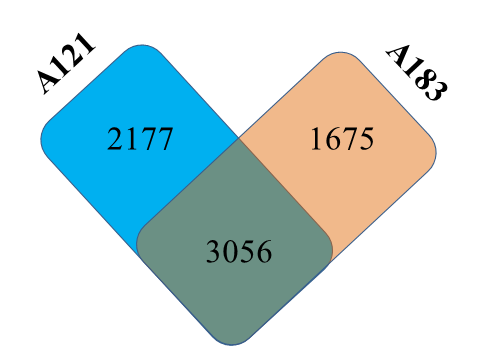


**Supplementary Figure 5** Venn diagram representing the number of shared and unique homologous gene clusters between the genomes.


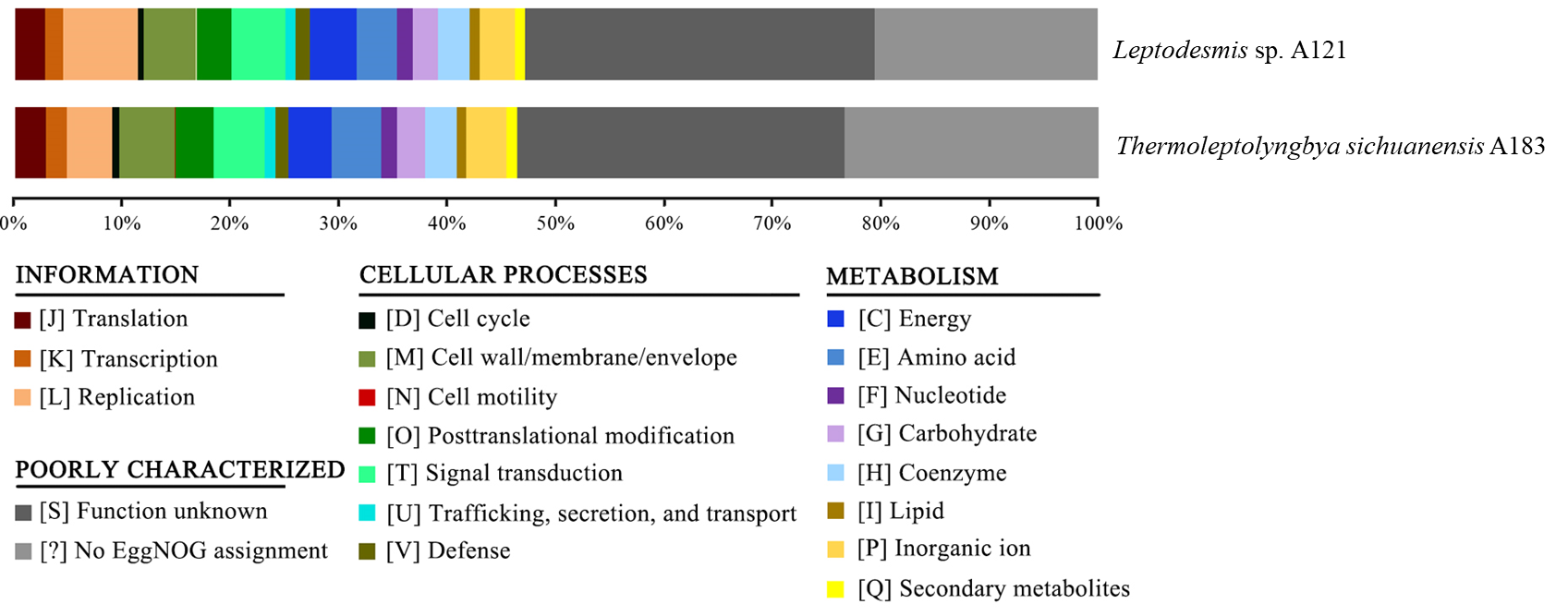


**Supplementary Figure 6** Functional classifications of protein-coding genes according to EggNOG database.


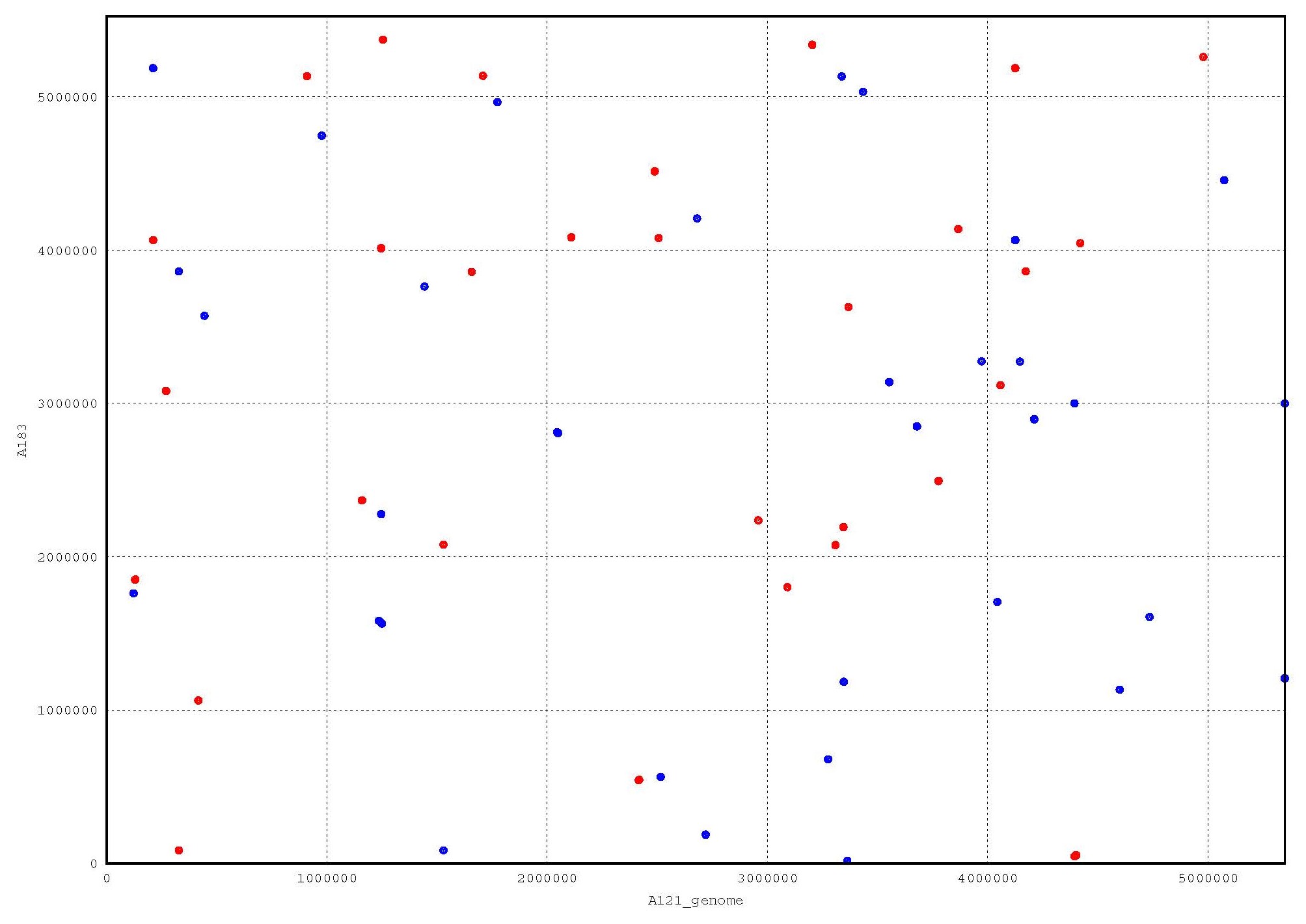


**Supplementary Figure 7** Pairwise genome alignments. The *Leptodesmis* sp. A121 genome was used as a reference for pairwise alignment with *Thermoleptolyngbya sichuanensis* A183 genomes. Red dots refer to positive matches, while blue dots represent reverse matches.


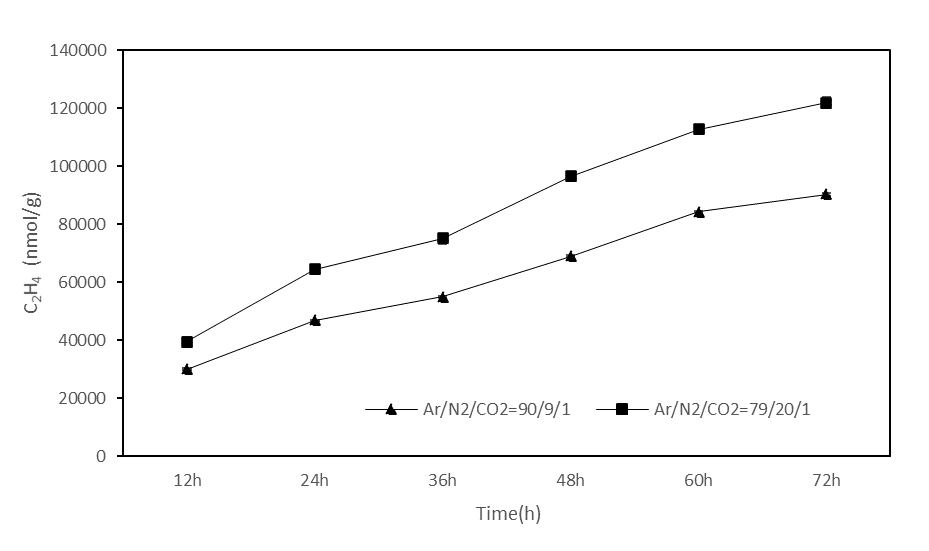


**Supplementary Figure 8** Nitrogenase activity of cell suspensions of *Leptodesmis sp.* A121 represented as a acetylene reduction assay. The figure presents ethylene formation in nmol/g_cells_ from nitrogen-free grown A121 cells during 72 h, 45℃, 30 μmol m-2 s-1 , gas composition Ar/N2/CO2 = 90/9/1 [v/v/v %] and Ar/N2/CO2 = 79/20/1 [v/v/v %]
